# Comparisons of Numerical and Experimental Investigations of the Thermal Performance of Al_2_O_3_ and TiO_2_ Nanofluids in a Compact Plate Heat Exchanger

**DOI:** 10.3390/nano12203634

**Published:** 2022-10-17

**Authors:** Wagd Ajeeb, S M Sohel Murshed

**Affiliations:** IDMEC, Department of Mechanical Engineering, Instituto Superior Técnico, University of Lisbon, 1049-001 Lisbon, Portugal

**Keywords:** plate heat exchanger, heat transfer, Al_2_O_3_ and TiO_2_ nanofluids, numerical model

## Abstract

This study reports the thermal performance of Al_2_O_3_ and TiO_2_ nanofluids (NFs) flowing inside a compact plate heat exchanger (CPHE) by comparing the experimental and numerical investigations. The NF samples were prepared for five concentrations each of Al_2_O_3_ and TiO_2_ nanoparticles dispersed in distilled water (DW) as a base fluid (BF). The stability of NF samples was ensured, and their viscosity and thermal conductivity were measured. Firstly, the experimental measurements were performed for the heat transfer and fluid flow of the NFs in the plate heat exchanger (PHE) system and then the numerical investigation method was developed for the same PHE dimensions and operation conditions of the experimental investigation. A finite volume method (FVM) and single-phase fluid were used for numerical modelling. The obtained experimental and numerical results show that the thermal performance of the CPHE enhances by adding nanoparticles to the BFs. Furthermore, numerical predictions present lower values of convection heat transfer coefficients than the experimental measurements with a maximum deviation of 12% at the highest flow rate. Nevertheless, the numerical model is suitable with acceptable accuracy for the prediction of NFs through PHE and it becomes better for relatively small particles’ concentrations and low flow rates.

## 1. Introduction

In the last decades, a strong trend in the industry was shown toward miniaturization and natural resource management, mainly the energy and materials sources (enhancing the energy efficiency of the systems and reducing the equipment sizes are considered important roads to natural resource management reduce the demand for energy sources and minerals). In this, heat transfer systems are widely spread in industry applications, and the development of heat exchangers continues. The latter gave rise to advanced heat exchangers called ‘‘compact heat exchangers’’ involving compact plate heat exchangers (CPHEs) that contain channels with relatively small mean hydraulic diameters, presenting miniature dimensions but with higher heat transfer effectiveness [1,2]. The unique design of plate heat exchangers (PHEs) consists of several plates separating two different cold and hot fluids providing large heat transfer surfaces between them [3]. So far, several configuration types of PHEs were developed for enhancing the heat transfer effectiveness according to the requirements of industrial applications such as aircraft, electronics, chemicals, and other applications that contain cooling and heating equipment. The wavy shape of the plates in the chevron PHEs causes turbulent fluid flow inside the channels even for low Reynolds numbers (*Re*) [4], thus offering better heat transfer effectiveness compared to other normal heat exchangers [5]. However, micro/mini channels/passages that are used in the compact heat exchanger models provide bigger heat transfer surfaces but also higher pressure drops that need higher pumping power in the system [6]. Moreover, high heat loads are presented in those compact heat exchangers which require an innovative method for intensively absorbing the heat from the surfaces. For this purpose, recent research in the field of thermal management systems, namely in compact heat exchangers, indicates to NFs as superior thermal fluids for enhancing the intensification of the heat transfer method [7]. Various types of nanoparticles have been used to produce NFs by mixing them with conventional heat transfer fluids, presenting various characteristics [8,9]. Besides the developed thermal conduction property of the NFs [10,11,12], there is an increase in the viscosity levels too, which is not preferable for heat exchangers due to the higher pumping power required [10,11,12]. Nevertheless, NFs were recommended to be used for compact heat exchangers to improve their thermal characteristics [13]. Moreover, a considerable recommendation was given to Al_2_O_3_ nanoparticles for the preparation of NFs due to achieving good dispersion and improvements in the heat transfer effectiveness [14,15,16]. An empirical and numerical research by Awais et al. [17] for Al_2_O_3_ NFs flow in a heat sink heat exchanger reported a good improvement of 17% to the thermal performance of the heat sink. Moreover, Choi et al. [18] reported a 6.9% improvement in the thermal performance of the radiator by using Al_2_O_3_ NFs as a coolant instead of the BF for a high-level power system. Another study by Huang et al. [19] studied the performance of PHE with Al_2_O_3_ and MWCNT NFs and an inconsiderable heat transfer improvement for both NFs was reported in comparison with BF, i.e., water. Furthermore, an empirical study by Mare et al. [20] presented a better cooling performance for the used CNTs NF than Al_2_O_3_ NF for the fluid flow through PHE.

So far, numerical examination methods such as computational fluid dynamic (CFD) tools showed good flexibility and a big advantage to be used for studying the heat transfer characteristics of heat exchangers by different numerical modelling methods [21,22]. Yet, numerical modelling has been used to simulate the performance of NFs for heat transfer of NFs flow through uniform mini-channels [23] and micro-channels [24], and it showed respectable agreement with experimental measurements for several conditions such as non-Newtonian rheology behavior for the NFs [25]. In addition, different computational methods were presented in the literature to investigate the behavior of NF flows for different applications such as solar energy systems, electronics, and automotive [26,27]. Ahmed et al. [28] numerically tested the thermal performance of Cu NFs in the isothermally corrugated channel and a considerable heat transfer upgrading was reported by using NF instead of water for *Re* between 100 and 1000. Other researchers have numerically tested the performance of other types of NFs. For example, Shirzad et al. [29] investigated Al_2_O_3_, CuO and TiO_2_ NFs in PHE for *Re* between 1000 and 8000. In their investigation, while Al_2_O_3_ NF had the best heat transfer values for low *Re*, TiO_2_ NF had the better performance in heat transfer for high *Re*. Bahiraei et al. [30] numerically tested the flow of Al_2_O_3_ NFs in micro PHE and different shapes of particles were used at 1.0 vol.%. concentration and *Re* of 500. Platelet-shaped Al_2_O_3_ particles presented the best heat transfer rates. There is a numerical research study conducted by Tiwari et al. [31] on PHE works with CeO_2_ and Al_2_O_3_ NFs as homogeneous fluids using the CFD tools (ANSYS-FLUENT). The numerical predictions were well matched with the experimental measurements and better performance was found for CeO_2_ NFs as coolants. Generally, preparation and stability influence the thermal performance in any heat transfer systems, such as PHE [7] and heat pipes [32].

In addition, the literature shows a single-phase numerical technique as a common method to simulate the NF’s behavior in heat exchangers and a good agreement is usually presented when numerical predictions are compared with experimental measurements [7]. Nevertheless, reported numerical investigations on NFs for compact heat exchangers were not supported by enough validation and comparison with experimental measurements [33,34], and the thermophysical properties of the used NFs are mostly obtained theoretically by using mixture laws and without studying the stability of NFs. Furthermore, the real mechanism behind the deviation between the numerical and experimental measurements was not identified, and the thermal behavior of the NFs in the flows was not explained. Therefore, the current study intended to perform firstly a thorough experimental determination of the thermophysical properties of Al_3_O_2_ and TiO_2_ NFs with low particle concentrations (0.01–0.2 vol.%) to be suitably tailored in the numerical simulations through the corrugated channel of compact chevron PHE. Furthermore, a careful experimental investigation was carried out for the heat transfer of the NFs flows in the hot loop of a compact PHE system. The details of the numerical methodology (geometry dimensions, boundary conditions, flow rates, etc.) were determined based on the experimental investigation and the equivalent working conditions were applied for an accurate comparison between the numerical and experimental measurements.

## 2. Nanofluids Preparation and Properties Characterization

In this study, a two-step preparation method was followed to produce the Al_2_O_3_ and TiO_2_ NFs samples. The primary sizes (diameter) of both Al_2_O_3_ and TiO_2_ nanoparticle were <50 nm and 20 nm, respectively, and their purity was 99.5% (as provided by IoLiTec, Heilbronn, Germany). The nanoparticles were dispersed into the BF (DW) for the volumetric concentration of 0.01, 0.05, 0.1, 0.15, and 0.2 vol.% (equivalent to 0.04, 0.2, 0,4, 0.6, and 0.8% of the mass fractions). First, nanoparticles were precisely weighed using a KERN ABS 80-4N scale and then dispersed into the base fluid. The good dispersion of nanoparticles into the base fluid was obtained after employing a magnetic stirring process for 15 min in a first moment followed by an ultrasonication process for 25 min using a probe-type ultrasonicator (Hielscher UP200Ht) at an amplitude of 60%, power of 110 W and 40 kHz frequency, to improve the dispersion and stability of the nanoparticles into the fluids. Moreover, the viscosity and thermal conductivity (*k*) of the NFs were directly measured after the preparation to ensure very good stability during the measurements. The higher thermophysical properties of NFs, mainly the thermal conductivity and viscosity, are considered key factors that define the heat transfer characteristics when NFs are employed to flow through the heat exchangers channels/passages. Therefore, these thermophysical properties of the NF samples were carefully measured and evaluated in the following sections. On other hand, specific heat (*C_p_*) and density of NFs do not change significantly at low particle concentrations, and mixture rules are widely used for the determination of these properties based on the volume fraction of particles (φ) into the BF. Thus, the mixture rules (e.g., [35,36]) which were applied to NFs are given by Equation (1) for density and Equation (2) for *C_p_*, respectively,

(1) $$\rho_{nf}=\varphi \rho_{p}+\left(1-\varphi \right)\rho_{bf}$$

(2) $$c_{p,nf}=\frac{\varphi {\left(\rho C_{p}\right)}_{p}+\left(1-\varphi \right){\left(\rho C_{p}\right)}_{bf}}{\rho_{nf}}$$

where the subscripts nf represent NF, p the particle, and bf the BF.

### 2.1. Thermal Conductivity

**Figure 1:**
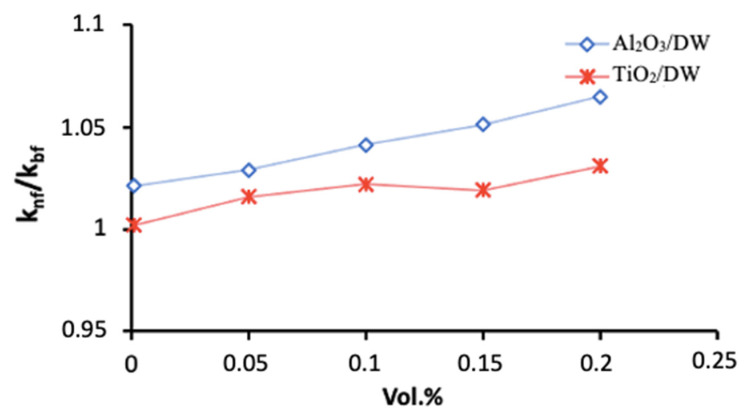
The enhancements of thermal conductivity of NFs as a function of volumetric concentrations of Al_2_O_3_ and TiO_2_ nanoparticles.

In this study, the thermal conductivity of the Al_2_O_3_ and TiO_2_ NFs was tested at room temperature (20 °C) by the transient hot-wire technique which showed good reliability for NFs measurements [37]. Several tests were performed for each NF with an interval time of 20 min. The resulted thermal conductivity’ values of the Al_2_O_3_ and TiO_2_ NFs are given in Figure 1 for several particles’ concentrations, and they show good enhancements up to 4.25% and 7.34% for TiO_2_ NF and Al_2_O_3_ NF at 0.2% particles’ concentration, respectively, compared to the BF. Moreover, Al_2_O_3_ NFs reported higher enhancement in the thermal conductivity values compared to TiO_2_ NFs at several values of φ. This is understandable as the thermal conductivity of Al_2_O_3_ has several times (~5 times) larger than that of the TiO_2_.

### 2.2. Viscosity

The rheology of NFs is considered a unique parameter for understanding their hydraulic and thermal behavior in the flows through heat exchanges. Therefore, the viscosity and rheology of the NFs and BFs are determined using a rheometer (from Brookfield) with a thermostatic bath for several temperatures and shear rates. However, the viscosity values did not change with shear rate values for all the types of particles and their amounts into the BF, which indicates a Newtonian behavior for those NFs. The latter (Newtonian behavior) was also reported in the literature for similar NF samples [38,39].

**Figure 2:**
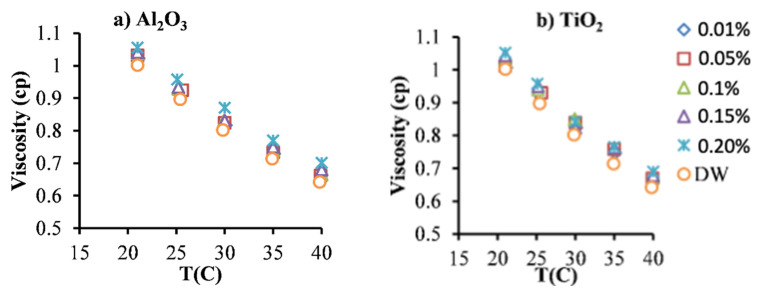
Viscosity of NFs as a function of temperature for two types of nanoparticles: (**a**) Al_2_O_3_, and (**b**) TiO_2_.

On the other hand, the viscosity’ values of Al_2_O_3_ and TiO_2_ NFs are tested for several temperatures’ values and φ. The resulted values are presented in Figure 2 show that the level of viscosity decreased by increasing the temperature value and raised by the increase in the particles’ volume fraction for both types of NFs (Al_2_O_3_ and TiO_2_ NFs), in similar behavior to most results in the literature [11,38].

It can be noticed a close values of viscosity for TiO_2_ and Al_2_O_3_ at the same particles’ concentrations and an increase of about 2.15% for 0.01 vol.% and of about 6.54% for 0.2 vol.% in comparison with the BF. Moreover, the decrease in the viscosity’ value due to the rise in the temperature is significant: up to around 33.6% for 0.2 vol.% when the temperature was increased from the lowest value of 21 °C to the highest value of 40 °C. The latter findings (viscosity results) were anticipated and agreed with the data in the literature for similar NFs [11,38].

## 3. Experimental Heat Exchanger System and Methods

**Figure 3:**
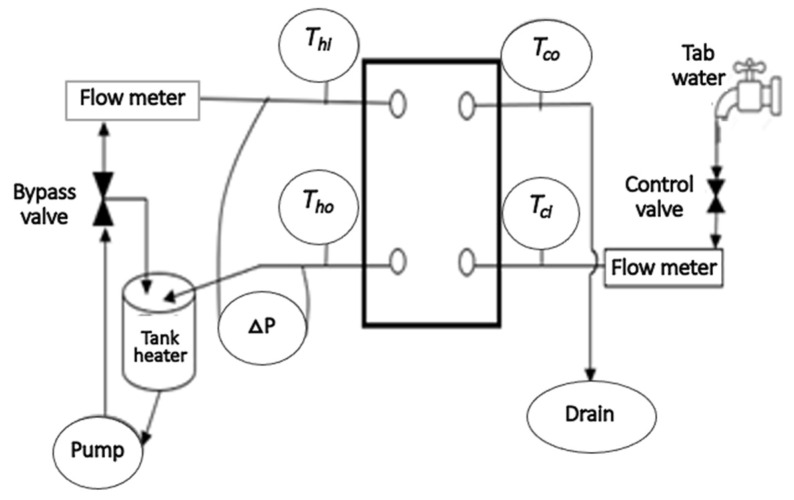
A flow diagram of the setup used for the experimental investigation.

The experimental rig of the CPHE was established for the fluid flow and heat transfer of nanofluids as presented in Figure 3. The CPHE system contains an open loop for the cold fluid (using only water that goes to the drain after passing the CPHE) and a hot fluid loop for the NF flow, which includes a tank with heater, pump, 2 flow meters, differential pressure sensor, 4 thermocouples for temperature measurements, and DAQ linked to PC for collecting data. Details about this investigational setup and working principle can be found in an earlier study [40]. The components’ accuracy of the investigational setup was firstly checked with DW as a well-known fluid and calibration procedure for the CPHE system was conducted [40]. A temperature of 40 °C was set for the NF at the inlet of the CPHE. The stabilization of the set flows and temperature were insured before recording the data by the data acquisition system considering 2 s interval time. In short, the fluids and NFs samples are heated in a tank then they flow into the hot loop passing the flowmeter and the CPHE to return to the tank. The temperatures of the fluid at the inlets and outlets of the CPHE are determined through four thermocouples and used to calculate the convection heat transfer coefficient (CHTC) for each sample at each flow rate.

The heat absorbed from the hot loop (*Q_h_*) and moved to the cold loop (*Q_c_*) is determined by Equations (3) and (4), and the average heat *Q* is assessed by Equation (5).

(3) $$Q_{h}={\dot{m}}_{h}C_{p,h}\left(T_{hi}-T_{ho}\right)$$

(4) $$Q_{c}={\dot{m}}_{c}C_{p,c}\left(T_{ci}-T_{co}\right)\;$$

(5) $$Q=(Q_{h}+Q_{c})/2$$

$T_{hi}$ and $T_{ho}$ represent the temperatures values at the inlet and outlet of the hot loop of the PHE, respectively. In addition, $T_{ci}$ and $T_{co}$ represent the temperatures values at the inlet and outlet of the cold loop of the PHE, respectively. Moreover, the overall convection heat transfer coefficient (*U*) is found by Equations (6) and (7).

(6) $$U=\frac{Q}{A\cdot LMTD}$$

*A* is the convection heat transfer area and *LMTD* is the log mean temperature difference.

(7) $$LMTD=\frac{\left(T_{ho}-T_{ci}\right)-(T_{hi}-T_{co})}{ln\frac{\left(T_{ho}-T_{ci}\right)}{\left(T_{hi}-T_{co}\right)}}\;$$

Then, the convection heat transfer coefficient (CHTC) for NFs in the hot loop ($h_{h}$) is defined by Equation (8):

(8) $$U=\frac{1}{h_{h}}+\frac{\delta }{k_{pl}}+\frac{1}{h_{c}}\;$$

where δ is the thickness of the plate of the heat exchanger, $k_{pl}$ represents the thermal conductivity of the plate’s material. Furthermore, $h_{c}$ is the CHTC for the water in the cold loop and it is theoretically predicted based on the heat exchanger design by Equation (9) [41] that has been used and validated in previous studies [42,43] for similar conditions.

(9) $$Nu=0.348Re^{0.663}pr^{0.33}$$

Then $h_{c}$ is determined from the definition of *Nu* as given by Equation (10),

(10) $$h_{c}=k\times Nu/D_{h}$$

*D_h_* is the hydraulic diameter of the channel in the CPHE ($D_{h}=2b$).

## 4. Numerical Modelling

The numerical investigation methodology was developed using the CFD tools of the ANSYS-FLUENT software package [44]. The numerical modelling was created based on the physics principles and the investigational procedure of the problem. The NFs samples were deemed single-phase fluids and the FVM method was employed. The previously determined (reported in the previous section) thermophysical properties of the NF samples were inserted into the numerical model corresponding to each NF sample. Moreover, a second-order upwind method is chosen for the convection and diffusing matters. The pressure and velocity of each fluid flow was linked by the SIMPLE method [45]. The simulations of the numerical approach are accomplished with achieving residual errors below 10^−6^ for the calculations of the governing equations.

### 4.1. Geometric Configuration and Boundary Conditions

**Figure 4:**
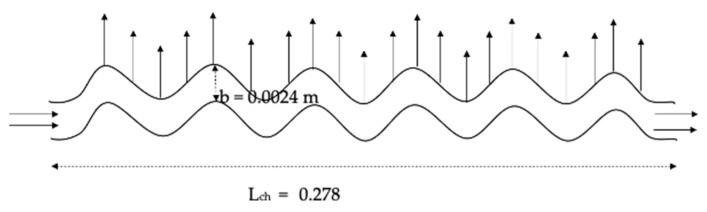
Schematic diagram of the numerical domain.

The dimensions of the numerical domain were chosen to approach and focus as much as possible on the physics of the problem of flows and heat transfer of the NFs in the PHE. Therefore, only the loop that contains the NFs’ flow was considered, while heat conditions of the cold loop (where the water is flowing at a constant flow rate during all the measurements) was obtained experimentally and applied in the numerical modelling into the boundary conditions of the NFs flows. The latter helps to avoid the possible numerical errors caused by the complex design of the PHE and reduces the time costs of the simulations by using a smaller number of nodes in the mesh of the numerical domain. Moreover, only one channel was chosen from the 5 hot channels in the hot loop for the simulations to increase the accuracy of the numerical investigations, mainly for NFs based on low particles concentrations. Therefore, the channel was designed to have similar dimensions to the channel of the PHE used in the investigational setup and the numerical domain was established as a two-dimensional (2D) corrugated channel with 0.278 m length (*L_ch_*) and 2.4 mm distance between channel’s plates (b) as it is presented in Figure 4. Moreover, the heat flux boundary conditions on the wall were found based on the measured data obtained for the cold loop, where the absorbed heat by the cold loop (*Q_c_*) was found by Equation (4). Thus, the heat absorbance value for one channel ($Q_{c1}$) can be calculated ($Q_{c1}=Q_{c}/5$), considering the assumption of the current study of having equal flows and heat rates in the five channels of the cold loop, then the heat flux per unit of area on the wall of the channel can be found in Equation (11).

(11) $$q=Q_{c}/A^{\prime }$$

where $A^{\prime }$ is the convection heat transfer area for one plate channel.

However, the numerical investigation methodology was established considering incompressible turbulent fluid flow. The thermophysical properties of the NFs and the BF were defined and used in the model. The inlet temperature (*T_in_*) was considered at 40 °C similar to the experimental measurement conditions for the hot loop. Moreover, velocity (*u*) at the channel inlet was determined based on the measurement procedures for each flow rate (0.03–0.93 L/s).

### 4.2. Governing Equations and Calculation

In this study, the equations of Navier–Stokes and energy are used in the numerical investigation methodology as governing equations for running the simulations for the NFs flowing through the corrugated channel of PHE. The turbulent model called as Realizable K-ε found in the FLUENT-ANSYS package is adapted, where it is considered a developed form of K-ε turbulence model proposed by Shih et at. [46] applying a recent formulation for eddy-viscosity.

The dimensional governing equations for the current study conditions are as follows in Equations (12)–(14),

Continuity equation:

(12) ∇·(ρV)=0

Momentum equation:

(13) ∇·(ρVV)=−∇P+∇·(μ∇V)

Energy equation:

(14) $$\nabla \cdot \left(\;\rho C_{p}VT\right)=\nabla \cdot \left(k\nabla T\right)$$

where V represents the vector of velocity, P is the pressure, ρ is the density of the fluid and μ is the viscosity of the fluid. Moreover, the kinetic energy of turbulence (*K*) and the dissipation rate (*ε*) are attained by the following transport equations (Equations (15) and (16)):

(15) $$\nabla \cdot \left(\rho VK\right)=\nabla \cdot \left[\left(\mu +\frac{\mu_{t}}{\sigma_{K}}\right)\nabla K\;\right]+G_{K}-\rho \varepsilon$$

(16) $$\nabla \cdot \left(\rho V\varepsilon \right)=\nabla \cdot \left[\left(\mu +\frac{\mu_{t}}{\sigma_{\varepsilon }}\right)\nabla \varepsilon \;\right]+\rho C_{1\varepsilon }S\varepsilon -\rho C_{2\varepsilon }\frac{\varepsilon^{2}}{K+\sqrt{\upsilon \varepsilon }}$$

In these equations, $G_{k}$ symbolizes the generation of turbulence kinetic energy term for the gradients in velocities. $C_{2\varepsilon }$ and $C_{1\varepsilon }$ are equation’ constants. $\sigma_{K}$ and $\sigma_{\varepsilon }$ represent the turbulent Prandtl numbers for K and ε, respectively. S is the average strain rate [44]. Moreover, the value of the eddy viscosity of the used Realizable model is not constant, and it is calculated from the following Equation (17):

(17) $$\mu_{t}=\rho \cdot C_{µ}\cdot \frac{K^{2}}{\varepsilon }$$

where $C_{µ}$ is a factor associated with the eddy viscosity. However, the model constants (C_2*ε*_, $\sigma_{K}$, *σ_ε_*, $A_{0}$ and $A_{s}$) have been formed to ensure the good performance of the numerical for the turbulent flow conditions. The mentioned constants of the numerical model are provided as:

$$C_{2\varepsilon }=1.90,\;A_{0}=4.040,\;\sigma_{K}=1.00,\;\sigma_{\varepsilon }=1.20\;\mathrm{and}\;A_{s}=\sqrt{6}\cos\varphi .$$

However, the simulations were carried out for the BFs and the NFs for the various particle concentrations (0.01, 0.05, 0.1 and 0.15 and 0.2 vol.%) flowing through the corrugated channel of the hot loop in the PHE in the similar working conditions (flow rates, temperature value, and thermophysical properties) of the experimental measurements. The temperature outcomes of the different samples at the outlet of the corrugated channel ($T_{ho}$) were collected and used to calculate the corresponding CHTC at each flow. The heat removed from the hot loop ($Q_{h}$) is found by Equation (3). Meanwhile, the values of the temperatures of the channel in a cold loop and the heat transferred ($T_{ci}$, $T_{co}$, and *Q_c_*) were already known from the experimental measurements. Then the mean heat transfer (*Q*) was calculated as in Equation (5). Moreover, the overall heat transfer coefficient (*U*) was found by Equations (6) and (7). Then, the CHTC for the BFs and NFs in the hot loop ($h_{h}$) can be determined by Equation (8). Where the $h_{c}$ is the CHTC for the water in the cold loop that was defined in the experimental investigation.

### 4.3. Mesh Optimisation and Validation

**Figure 5:**
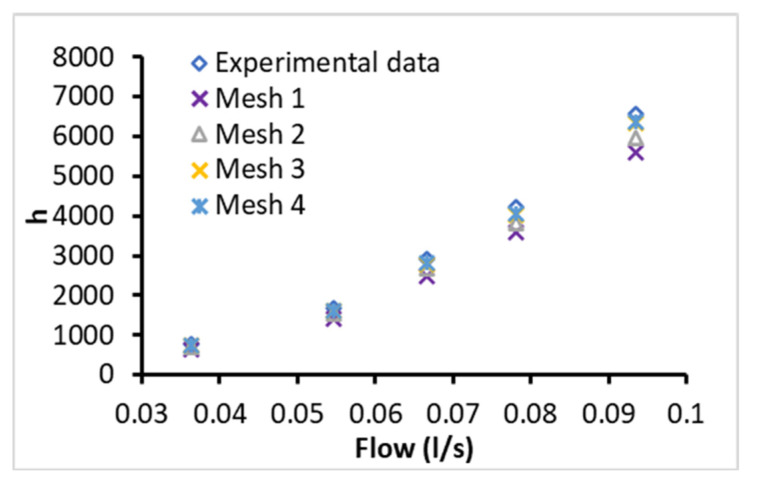
Mesh independency and validation for CHTC (*h*) of BF as a function of flow rate.

**Figure 6:**
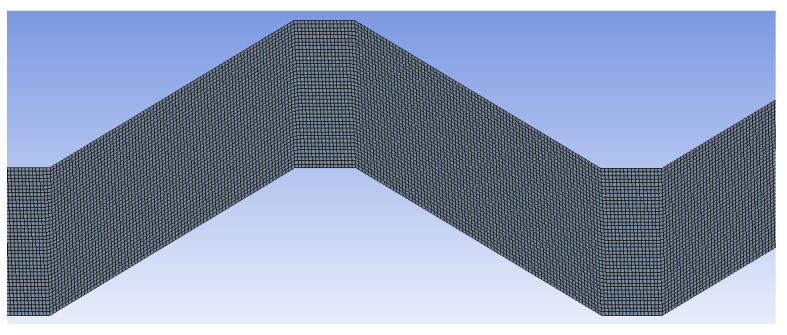
A view of a section of the mesh of the numerical domain.

The independency of the mesh was ensured by testing several meshes by increasing the number of the nodes and comparing it with experimental measurements for water as it has a well-known hydraulic-thermal behavior. The tested meshes were 112621 (Mesh 1), 168441 (Mesh 2), 219282 (Mesh 3), and 303101 (Mesh 4): nodes were tested for several flow rates at the equivalent working conditions of the experimental investigation. The obtained numerical predictions are presented in Figure 5 for the CHTC and they show good accuracy for the mesh of 219282 nodes in comparison with the experimental measurements (Experimental data) with a deviation of around 6%. Moreover, it was found that the numerical predictions of CHTC were not changing for node numbers higher than 219282 nodes. Therefore, the mesh of 219282 nodes was selected for the intended simulations of this study to ensure the independency of the results from the mesh. A part of the mesh is presented in Figure 6, which shows the uniform and smooth structure of the mesh for accurate simulations of the turbulent flows.

**Figure 7:**
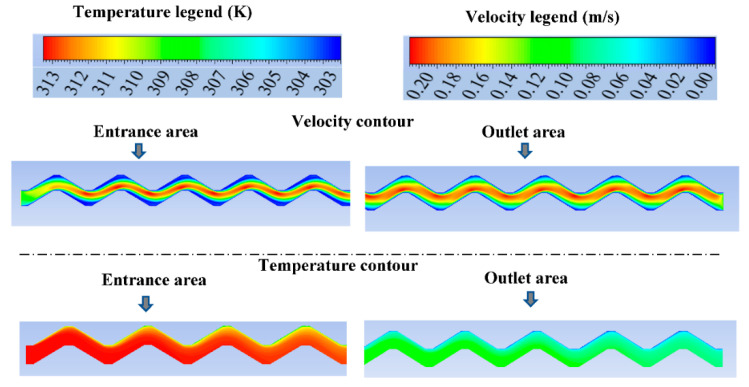
The numerical section of study (test) with the temperature and velocity profiles at the entrance and outlet of the channel.

Furthermore, the resulted profiles of the velocity and temperature fields, at the entrance and end of the channel of the PHE, are presented in Figure 7 which shows good consistency with the fluid flow characteristics under the PHE problem conditions. It can be noticed from Figure 7 that the values of the temperature at the entrance of the channel starts high and it becomes lower in the outlet area of the channel with lower values of temperature near the upper wall. Moreover, the velocity profile shows the boundary layer of the flow where there are lower values of velocity near the walls which make closely a parabolic profile of the velocity in the channel.

## 5. Heat Transfer Results and Discussion

**Figure 8:**
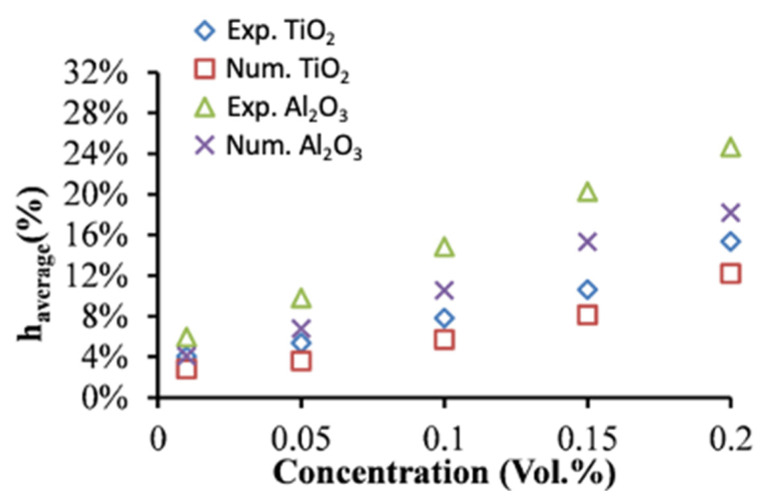
Enhancement of average heat transfer coefficient of DW-based Al_2_O_3_ and TiO_2_ NFs obtained experimentally and numerically as a function of nanoparticle concentration.

The experimental and numerical results of the heat transfer performance of two types of NFs (Al_2_O_3_ and TiO_2_ NFs) flowing through the hot loop of the CPHE are presented in Figure 8. The heat transfer enhancements of the NFs are determined in comparison with the corresponding BFs. The outcomes show good improvement with the increase in loading of nanoparticles for all flow rates (Figure 8), and better thermal performance for Al_2_O_3_ compared to TiO_2_ NFs for a deviation of 9% at 2.0 vol.% particles that decreases with reducing the concentration of the particles. The maximum average enhancement of about 24.6% is observed for the highest particle concentration (0.2 vol.%) through the experimental measurements (Figure 8) of Al_2_O_3_ NF. It was anticipated as the enhanced thermal conductivity of nanofluids was found to increase with increasing the amount of nanoparticles into the BF. Moreover, it is due to the fact that Al_2_O_3_ NFs exhibit higher thermal conductivity compared to TiO_2_ NFs (as presented in Figure 1).

Therefore, the numerical findings (Num. in Figure 8) and experimental measurements (Exp. in Figure 8) confirm that the dispersion of nanoparticles characterized by superior thermal conduction such as Al_2_O_3_ and TiO_2_ particles with a conventional heat transfer fluid (e.g., DW in this study) enhances the heat transfer performance, due to the greater thermal conductivity of the NFs. As presented in Figure 8, an increase in nanoparticles’ concentrations led to higher enhancement value in CHTC. It should be also noted that the temperature and velocity profiles and their boundary layer developments into the flow regime are influenced by the advanced properties of the NFs performing better heat transfer between the two loops of the PHE, especially in the conditions of the current study where the NFs are operating in the hot loop under high temperature that leads to having higher thermal conduction characteristic and lower viscosity values of the NFs. The latter explains the excellent heat transfer improvement that reached around 24.6% for 0.2 vol.% of Al_2_O_3_ and 15.3% for 0.2 vol.% of TiO_2_. On other hand, the random movement of the nanoparticle in the turbulent flow regime and the possible migration of the nanoparticles into the flow inside the PHE (as appeared in the experimental investigations) can cause further development in the heat transfer rates [47]. These mentioned factors can also be the reasons for the higher enhancements of the CHTCs for Al_2_O_3_ and TiO_2_ NFs than the enhancements of their thermal conductivity (e.g., Figure 1). Several relevant studies have also highlighted that the improved convection heat transfer for the laminar flow of Al_2_O_3_ NFs through a horizontal tube mainly due to the migration phenomena of nanoparticles into the flow [48]. The latter is proved by the current study when the experimental results were compared with the numerical results where the impact of the nanoparticles’ movements and the migration phenomena are not considered for the numerical approach (nanofluids are simulated as single-phase fluids) but it exists in the experimental investigation. However, the advanced heat transfer performance of Al_2_O_3_ NFs for different types of heat exchangers was widely reported in the literature [12,49,50] as well as for TiO_2_ NFs [51,52] in agreement with the finding of the current study. Furthermore, Tiwari et al. [42] found in their experimental investigation of CeO_2_, Al_2_O_3_, TiO_2_, and SiO_2_ NFs for gasketed PHE that there was heat transfer boost for all the particles’ types with maximum values for CeO_2_ NFs, whereas the Al_2_O_3_ NFs showed better heat transfer enhancement than TiO_2_ NFs. Nevertheless, the findings and discussion can vary among different researchers even for the same NF type because of many parameters related to the concentration and morphology of the particles, BF, flow rate, the operation temperature value, and the method of preparing the NFs and the type of heat exchanger and its dimensions.

**Figure 9:**
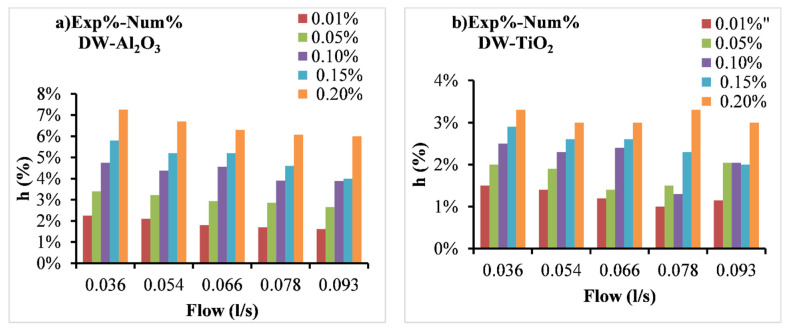
The deviation between experimental (Exp) and numerical (Num) findings of the enhancement of CHTC (h%) as a function of flow rate of (**a**) Al_2_O_3_ and (**b**) TiO_2_ NFs.

On the other hand, the differences between the numerical and the experimental results are shown in Figure 9. The numerical results show relatively lower heat transfer enhancements in comparison with experimental data with a deviation between 1.0% and 3.3% for TiO_2_ NFs and a deviation between 1.6% and 7.2% for Al_2_O_3_ NFs.

Moreover, the deviation, in most cases, increases with the decrease in flow rates and the rise in particles concentrations (Figure 9). The latter refers to the existence of some factors in experimental investigations responsible for extra heat transfer enhancement than the ones in numerical investigations. Those factors are mainly related to the nanoparticle’s movements in the flow which is not considered in the numerical investigation methodology. However, at higher flow rates the hydraulic impact of the flow on convection heat transfer performance becomes higher than the impact of the thermal conductivity increases in the NFs which led to a slightly lower impact of the nanoparticle’s movements on heat transfer. On other hand, the increase in the deviation between the numerical and experimental data refers to the increase in the impact of nanoparticles’ movements with increasing the concentration due to their influence on hydraulic and thermal boundary layer development leading to higher heat transfer levels. However, some previous studies have conducted both numerical and experimental examinations on the NFs through PHE, such as a study by Pantzali et al. [53] for CuO NFs in a miniature PHE and they mentioned better heat transfer levels at low flow rates reaching the overall heat transfer enhancement of about 29.41%. Their numerical results were in good agreement with the experimental results, demonstrating CFD as a reliable tool for investigating NFs in PHE. Moreover, Bhattad et al. [54] have numerically and empirically examined the behavior of hybrid NF (Al_2_O_3_ + MWCNT/water) in PHE and reported an increase in CHTC of 39.16%. Their numerical predictions were in good agreement with the experimental results with a smaller deviation. The latter conclusion is also agreed with the findings of a numerical study by Tiwari et al. [31] using CeO_2_ and Al_2_O_3_ NFs in PHE. Therefore, the numerical results of the current study showed significant advantages to define the parameters that cannot be determined when only the experimental methods are used. The parameters such as the nanoparticles movements and their impact on the fluid flow and heat transfer through the channels of PHE can’t be defined by the traditional experimental investigation methods. The numerical results of the current study allowed to isolate (comparing with the experimental data of heat transfer) those important parameters that are considered responsible for the extra enhancement of the heat transfer.

## 6. Conclusions

In this study, numerical simulations and experimental measurements on the flow and heat transfer performance of Al_2_O_3_ and TiO_2_ NFs in a compact PHE are carried out. The PHE system was established and verified for the operation of the flows of two fluids (cold and hot) in separated loops. The Al_2_O_3_ and TiO_2_ NFs are prepared and their thermophysical properties such as thermal conductivity and viscosity are measured. The improvements in thermal conductivity caused by adding different concentrations of nanoparticles are presented, and maximum enhancements of about 7.30% for Al_2_O_3_ NF and 4.20% for TiO_2_ NF at 0.2 vol.% concentration were found. Additionally, the viscosity was found to increase by increasing concentration of nanoparticles for both NFs, and there was a decrease with increasing the temperature. The numerical methodology is developed using the CFD tools of the ANSYS-FLUENT software for the similar physical conditions of the experimental method.

The heat transfer investigations on the Al_2_O_3_ and TiO_2_ NFs through the hot loop of the compact plate heat exchanger were conducted for several flow rates at an inlet temperature of 40 °C, and the heat transfer characteristics were empirically determined. The experimental and numerical data obtained for both NFs regarding CHTC enhancements were compared for several flows. Good enhancements of the heat transfer were found for both NFs and it was found to increase with the concentration of particles for all the flow rates. However, Al_2_O_3_ NFs showed better enhancement compared to TiO_2_ NFs. The maximum enhancement of heat transfer (24.6%) was observed for the Al_2_O_3_ NF at the highest particle concentration (0.2 vol.%) through the experimental measurements.

Moreover, the numerical results show lower heat transfer enhancements in comparison with the experimental measurements with a deviation between 1.0% and 3.3% for TiO_2_ NFs and a deviation between 1.6% and 7.2% for Al_2_O_3_ NFs. The deviation was changed based on the particles’ concentration and the flow rate. The latter is presumed due to the nanoparticles’ movements in the flow which is not considered in the numerical investigation and led to extra heat transfer enhancement.

Finally, the experimental and numerical findings of flows of Al_2_O_3_ and TiO_2_ NFs showed good heat transfer enhancements in the compact PHE. This study helps for a better understanding of the heat transfer performance and mechanisms of the NFs’ behavior through such PHE as well as highlights the benefits of using CFD tools for modelling NFs with clarification of their thermal characteristics.

## Nomenclature

Al_2_O_3_	Alumina
b	Distance between the channels’ plates (mm)
BF	Base Fluid
*C_p_*	Specific heat of the fluid (J/kg⋅K)
CFD	Computational Fluid Dynamic
CPHE	Compact plate heat exchanger
$D_{h}$	Hydraulic diameter of the channel (m)
DW	Distilled Water
*h*	Convection Heat transfer coefficient (W/m^2^⋅K)
CHTC	Convection Heat transfer coefficient (W/m^2^⋅K)
*k*	Thermal conductivity of the fluid (W/m⋅K)
LMTD	Log mean temperature difference
$\dot{m}$	Mass flow (kg/s)
NF	Nanofluid
PHE	plate heat exchanger
*Q*	Heat rate (W)
T	Temperature (K)
*U*	Overall convection heat transfer coefficient (W/m^2^⋅K)
Greek symbols	
μ	The viscosity (mPa⋅s)
ρ	The density (kg/m^3^)
δ	Plate thickness (mm)
φ	Volume fraction of particles
Subscripts	
*bf*	Base fluid
*c*	Cold
*f*	Fluid
*h*	Hot
*i*	Inlet
*nf*	Nanofluid
*o*	Outlet
*p*	Particle
*pl*	Plate
